# Theoretical impact of simulated workplace-based primary prevention of carpal tunnel syndrome in a French region

**DOI:** 10.1186/s12889-018-5328-6

**Published:** 2018-04-02

**Authors:** Yves Roquelaure, Natacha Fouquet, Emilie Chazelle, Alexis Descatha, Bradley Evanoff, Julie Bodin, Audrey Petit

**Affiliations:** 1 Irset (Institut de recherche en santé, environnement et travail), UMR_S 1085, University of Angers, CHU Angers, University of Rennes, Inserm, Ehesp, F-49000 Angers, France; 2 Santé publique France, Equipe associée en Epidémiologie et Prévention des TMS (EpiPrevTMS), F-94415 Saint-Maurice, France; 3 INSERM UMS 011, Population Based Epidemiological Cohorts Unit and University Versailles St-Quentin, F-78035 Versailles, France; 4 Division of General Medical Sciences, Washington University School of Medicine, St. Louis, MO 63310 USA

**Keywords:** Carpal tunnel syndrome, Work-related, Attributable risk, Prevention, Simulation, Preventive efficiency

## Abstract

**Background:**

Carpal tunnel syndrome (CTS) is the most common nerve entrapment neuropathy in the working-age population. The reduction of CTS incidence in the workforce is a priority for policy makers due to the human, social and economic costs.

To assess the theoretical impact of workplace-based primary interventions designed to reduce exposure to personal and/or work-related risk factors for CTS.

**Methods:**

Surgical CTS were assessed using regional hospital discharge records for persons aged 20–59 in 2009. Using work-related attributable fractions (AFEs), we estimated the number of work-related CTS (WR-CTS) in high-risk jobs. We simulated three theoretical scenarios of workplace-based primary prevention for jobs at risk: a mono-component work-centered intervention reducing the incidence of WR-CTS arbitrarily by 10% (10%-WI), and multicomponent global interventions reducing the incidence of all surgical CTS by 5% and 10% by targeting personal and work risk factors.

**Results:**

A limited proportion of CTS were work-related in the region’s population. WR-CTS were concentrated in nine jobs at high risk of CTS, amounting to 1603 [1137–2212] CTS, of which 906 [450–1522] were WR-CTS. The 10%-WI, 5%-GI and 10%-GI hypothetically prevented 90 [46–153], 81 [58–111] and 159 [114–223] CTS, respectively. The 10%-GI had the greatest impact regardless of the job. The impact of the 10%-WI interventions was high only in jobs at highest risk and AFEs (e.g. food industry jobs). The 10%-WI and 5%-GI had a similar impact for moderate-risk jobs (e.g. healthcare jobs).

**Conclusion:**

The impact of simulated workplace-based interventions suggests that prevention efforts to reduce exposure to work-related risk factors should focus on high-risk jobs. Reducing CTS rates will also require integrated strategies to reduce personal risk factors, particularly in jobs with low levels of work-related risk of CTS.

## Background

Carpal tunnel syndrome (CTS) is the most common nerve entrapment neuropathy in the working-age population and one of the most common causes of workers’ compensation claims worldwide [1, 2].

Numerous risk factors for CTS have been identified, some relating to personal characteristics and other to work-related biomechanical constraints. Certain personal susceptibility attributes (e.g. age, gender, wrist shape, genetics) cannot be modified by prevention interventions and/or medical interventions [3, 4], in contrast to some modifiable systemic conditions (e.g., obesity, diabetes mellitus, arthritis) [4–6] and habits (e.g., smoking, domestic work, physical exercise) [7]. Exposure to work-related biomechanical risk factors for CTS (e.g., repetitive movements, hand-arm transmitted vibration, forceful manual exertion, and bending/twisting of the wrist) can be modified by workplace-based interventions [2, 8–10], as can exposure to work-related psychosocial risk factors for CTS [8, 11].

The multifactorial origin of CTS makes it difficult to distinguish the relative contribution of personal and work-related factors at the individual level. However, at the population level a substantial number of CTS are mainly related to workers’ personal characteristics and medical conditions. Cases that occur regardless of work exposures will be called ‘personal-related CTS’ (PR-CTS) in the remaining part of the text. Other cases occurring in excess in workers employed in jobs at high risk for CTS can be considered as mainly work-related or ‘attributable to work’. The proportion of ‘work-related CTS’ (WR-CTS) can be estimated by the work-related attributable fraction of risk (AFE).

The reduction of CTS incidence in the workforce is a priority for policy makers due to the human, social and economic costs. Work-centered ergonomic interventions (WI) for primary prevention include ergonomic and organizational adaptation of the workplace (provision of equipment reducing physical exposures, ergonomic workplace design, optimization of work organization, organizational development, participatory ergonomics, etc.). Some multifaceted global interventions (GI) add to the WI component various components of personal interventions (PI), such as worksite behavioral programs (e.g., social health promotion, wrist stretching exercise), education programs (e.g., education and training on risk-reducing working techniques) and diet programs to manage overweight [12, 13]. Pre-employment examination is sometimes used as a strategy to reduce WR-CTS, however this practice is ineffective [14].

Multi-component global interventions (GI) including both personal behavioral interventions (PI components) and collective technical, ergonomic and organizational interventions (WI components) are considered the most promising preventive approach for upper extremity musculoskeletal disorders among workers [15–20]. However, information is still scant on their sustainability and effectiveness to reduce CTS risk factors (primary prevention) and/or the duration of symptoms and sickness absence following CTS (secondary/tertiary interventions) [15–19, 21, 22]. To the best of our knowledge, information is still lacking concerning the effectiveness of WI and GI interventions to reduce the incidence of CTS [23].

From a theoretical point of view, WIs focusing on work-related risk factors are expected to reduce mainly ‘WR-CTS’; their impact will depend on the proportion of cases attributable to work. Using surveillance data in the general population of the French Pays de la Loire region, we estimated work-related AFE ranging from 19% in female blue collar- workers, 24% in female low grade white collar workers to 50% in male blue collar workers [24]. This suggests that up to 19–50% of incident cases of CTS occurring in these workers could be avoided if effective intervention programs were available and implemented in high-risk occupational categories and industry sectors. Higher values can be expected for interventions focusing on some jobs at particularly high risks (high AFEs). Primary multi-component global interventions (GI) are expected to be the most efficient in targeting both ‘PR-CTS’ and ‘WR-CTS’, regardless of the AFE value [20, 25]. However, we still lack of information on the joined effects of reducing occupational and non-occupational CTS risk factors when present in combination.

### Aims

In lack of controlled intervention studies comparing the effectiveness of workplace-based WI and GI [16, 26], information on AFE could be useful to estimate the theoretical preventive impact of workplace-based primary prevention interventions aiming to reduce exposure to work-related and/or personal risk factors for CTS. The aims of this pilot study were therefore to estimate the number of cases of surgically-treated CTS (CTS) attributable to work in jobs at high risk in the population of working age, and to simulate the impact of workplace-based mono-component WI and multi-component GI preventive primary interventions aiming to reduce the incidence of CTS.

## Methods

Since 2002, the French national public health agency, Santé publique France, has conducted an epidemiological surveillance program of CTS in the Pays de la Loire region using multiple data sources. This program has been described in detail elsewhere [27]. Only information on surgically treated CTS (WHO ICD-10 code G 56.0) was considered in this study.

### Population

The population included in this surveillance program was made up of residents of the Pays de la Loire region (Loire Valley area, west central France) in the 20–59 age group (914,999 women and 914,957 men), whether they were professionally active or not, in 2009. According to the 2009 census data, the region has 5.7% of the French population, with a diversified socioeconomic structure and high employment rate (70.0% for women and 76.4% for men) [28].

### Outcomes

The hospital discharge database of the French National Medical Information Systems Program (PMSI) was analyzed to include all patients aged 20 to 59 years residing in the region and having undergone surgery for CTS (WHO ICD-10 code G 56.0) in 2009.

### Occupational history

Due to a lack of information on employment status in the PMSI database, we used data collected by a pilot study conducted among 1500 persons (1053 women and 447 men) having undergone CTS surgery in the three largest hand surgery settings of the region in 2002–2003 (unpublished data). Employment status and jobs at the time of surgery were experimentally registered in the medical files of the hand clinics during the pilot study and were available for 1371 (91%) patients (975 (92.6%) women and 396 (88.6%) men): 1149 (83.8%) were professionally active (784 (80.4%) women and 365 (92.2%) men) and 222 (19.3.8%) were inactive (191 (19.6%) women and 31 (7.8%) men). Occupations were coded using the French version of the Classification of Economic Activities in the European Community (Nace codes) and the French classification of occupations (PCS codes). Jobs were identified by a combination of the occupational category and the industry sector using the 2-digit PCS and 2-digit Nace codes.

### Scenarios of prevention

In absence of precise data in the literature, we arbitrarily hypothesized that interventions could reduce the incidence of CTS by 10% in high-risk jobs, and simulated three scenarios of workplace-based primary prevention, differing by their main target:

- *10% WI*: mono-component work-centered intervention targeting only work-related risk factors for CTS (e.g., ergonomic intervention: workstation redesign, establishment of an ergonomics task force, job rotation, ergonomics training, etc.) expected to reduce WR-CTS by 10%;
- *5%-GI* and *10% GIs:* multi-component global interventions targeting both personal and work-related risk factors for CTS and expected to reduce both PR-CTS and WR-CTS by 5% or 10%, respectively.

### Statistical analysis

Incidence rates of CTS in the whole population (*I*_*wp-cts*_) were computed separately for each gender using the regional 2009 PMSI database and 2009 census data. Using the information from the 2002–2003 pilot study, three indicators were computed. 1) The age-adjusted standardized incidence ratios of CTS *(SIR*_*job-cts*_*)* were calculated for each job with all other jobs as reference. 2) The age-adjusted relative risks of CTS according to job (*aRR*_*job-cts*_) were computed using the Mantel-Haenszel method, with the whole sample of subjects included in the study as reference, whether they were employed at the time of diagnosis or not. 3) The contribution of the jobs to the occurrence of CTS was quantified with the age-adjusted attributable fraction of risk in exposed individuals (AFE) which estimates the proportion of surgical CTS attributable to work in the jobs at high risk of CTS [24, 29]: *AFE*_*job-cts*_ *= (aRR*_*job-cts*_*-1) / aRR*_*job-cts*_.

These indicators were computed for each job when (i) more than 15 men or women were employed and (ii) *aRR*_*job-cts*_ was significantly higher than 1. Jobs at high risk of CTS in comparison with the whole population were thus detected and called “high-risk jobs”. Then, specific incidence rates (*I*_*job-cts*_) were computed according to high-risk jobs (the numerators were obtained by multiplying ‘*I*_*wp-cts*_’ by the SIR of CTS in the considered job, the denominators were the number of persons of the same gender in the job). The total number of CTS *(N*_*job-cts*_*)* in the job considered was computed by multiplying the number of workers employed in this job (N_e-job_) by the incidence rate in this job (*I*_*job-cts*_):

*N*_*job-cts*_ *= N*_*e-job*_ x *I*_*job-cts*_*.*

The number of WR-CTS *(N*_*job-wr-cts*_*)* was calculated by multiplying the total number of CTS by the AFE in the job considered [29]:

*N*_*job-wr-cts*_ *= N*_*job-cts*_ x *AFE*_*job-cts(%)*_*.*

The preventive efficiency (PE) was estimated as the ratios of CTS hypothetically avoided / total number of CTS (%) in the job considered. A 95% confidence interval was computed only for *SIR*_*job-cts*_ and for *aRR*_*job-cts*_. For other indicators, a range was calculated using the lower and upper limits of the considered indicator in the calculation formula.

## Results

In 2009, 5469 hospital discharges for median nerve release at the wrist were registered for the regional general population aged 20–59 (3846 in women and 1623 in men). The annual incidence rate of surgical CTS per 1000 person-years (*I*_*wp-cts*_) was 3.0 for both genders (4.2 in women and 1.8 in men).

### Estimated number of cases of CTS in the high-risk jobs

A total of nine jobs were identified as being at high risk of CTS in women (7 jobs) and/or men (4 jobs) accounting for 15% of the region’s workforce (women, 23%; men, 9%) (Table 1). Five jobs were at risk only in women: government and public service personal care workers (e.g., aide-nurses) in the health sector (‘Healthcare jobs’), sales workers and cashiers in the trade and commerce sectors (‘Trade jobs’), personal service workers (e.g., home aides) in the health sector in women (‘Personal services health jobs’), personal service workers (e.g., housekeepers) and cleaners in the services to enterprises sectors (‘Cleaning jobs’) and machine operators and assemblers in the shoe industry sector (‘Shoe industry jobs’).

**Table 1:** Standardized incident ratios (SIR) and incidence of carpal tunnel syndrome (CTS), attributable risk fractions among the exposed (AFE) and estimated number of cases of CTS in the high-risk jobs in men and/or women

	*N*_Workers_^m^	% _Workers_^a^	SIR_job_^b^	95%	CI^c^	I_CTS_^d^	Range^e^		AFE ^f^	Range^g^		*N*_*job-cts*_^h^	Range^i^		*N*_WR-CTS_^j^	Range^k^	
‘Agriculture jobs’																	
Women^l^	5077	0.7	3.8	2.9	5.0	16.1	12.0	21.2	77.0	69.5	82.6	82	61	108	63	42	89
Men^l^	9907	1.3	2.0	1.2	3.1	3.5	2.1	5.5	64.5	42.0	78.3	34	20	54	22	8	42
Total	14,984	1.0										116	81	162	85	50	131
‘Food industry jobs’																	
Women^l^	7442	1.1	4.9	3.1	7.2	20.4	13.2	30.2	83.0	74.7	88.6	152	98	225	126	73	199
Men^l^	7370	0.9	6.0	3.4	9.7	10.6	6.1	17.2	88.3	80.7	92.9	78	45	127	69	36	118
Total	14,811	1.0										230	143	352	195	109	317
‘Shoe industry jobs’																	
Women^l^	1305	0.2	2.9	2.0	3.9	12.1	8.6	16.5	71.0	60.1	78.9	16	11	22	11	7	17
Men	381	0.0	1.7	0.5	3.9	3.0	1.0	7.0	71.7	31.6	88.3	1	0	3	1	0	3
Total	1686	0.1										17	11	25	12	7	20
‘Construction jobs’																	
Women	610	0.1	8.7	0.2	48.3	36.5	0.9	203.1	93.8	60.7	99.0	22	1	124	21	1	123
Men^l^	31,329	4.0	2.6	1.8	3.5	4.5	3.2	6.2	65.5	51.8	75.4	142	100	196	93	52	148
Total	31,939	2.1										164	101	320	114	53	271
‘Transport jobs’																	
Women	2691	0.4	2.1	0.3	7.8	9.0	1.1	32.6	88.8	55.6	97.2	24	3	88	21	2	86
Men^l^	18,955	2.4	2.5	1.5	4.0	4.4	2.6	7.0	69.5	51.0	81.0	84	50	133	58	25	108
Total	21,646	1.4										108	53	221	79	27	194
‘Cleaning jobs’																	
Women^l^	7434	1.1	3.2	1.8	5.1	13.3	7.7	21.3	74.3	58.6	84.1	99	58	158	74	34	133
Men	5671	0.7	0.6	0	3.6	1.1	0	6.3	71.0	0	96.0	6	0	36	4	0	35
Total	13,105	0.9										105	58	194	78	34	168
*Group 1*																	
*Women*	*24,559*	*3.5*										*395*	*232*	*725*	*316*	*159*	*647*
*Men*	*73,613*	*9.3*										*345*	*215*	*549*	*247*	*121*	*454*
*Total*	*98,172*	*6.6*										*740*	*447*	*1274*	*563*	*280*	*1101*
‘Trade jobs’																	
Women^l^	36,197	5.1	1.8	1.3	2.5	7.6	5.3	10.5	53.6	35.1	66.8	274	192	379	147	67	253
Men	8323	1.1	0.9	0.1	3.3	1.6	0.2	5.9	76.2	4.1	94.1	14	2	49	11	0	46
Total	44,520	3.0										288	194	428	158	67	299
‘Healthcare jobs’																	
Women^l^	52,492	7.4	1.7	1.4	2.2	7.3	5.8	9.0	48.2	34.8	58.7	381	302	475	183	105	279
Men	6774	0.9	0			0			0			0			0		
Total	59,266	4.0										381	302	475	183	105	279
‘Personal services health jobs’																	
Women^l^	49,404	7.0	1.3	1.0	1.6	5.3	4.0	6.8	23.2	0.5	40.7	261	200	335	60	1	136
Men	913	0.1	3.9	0.1	21.6	6.9	0.2	38.3	90.3	33.1	98.6	6	0	35	5	0	35
Total	50,317	3.4										267	200	370	65	1	171
*Group 2*																	
*Women*	*138,093*	*19.5*										*916*	*694*	*1189*	*390*	*173*	*668*
*Men*	*16,011*	*2.0*										*20*	*2*	*101*	*16*	*0*	*81*
*Total*	*154,104*	*10.3*										*936*	*696*	*1290*	*406*	*173*	*749*
*All high-risk jobs*																	
*Women*	*159,351*	*22.5*	*2.0*	*1.8*	*2.3*	*8.5*	*7.6*	*9.5*	*59.9*	*54.1*	*64.9*	*1265*	*922*	*1702*	*664*	*329*	*1106*
*Men*	*67,561*	*8.5*	*2.7*	*2.1*	*3.3*	*4.7*	*3.8*	*5.8*	*68.1*	*59.7*	*74.8*	*338*	*215*	*510*	*242*	*121*	*416*
*Total*	*226,912*	*15.1*										*1603*	*1137*	*2212*	*906*	*450*	*1522*
*Whole population*																	
*Women*	*914,999*	*100*	*–*			*4.2*			*–*			*3846*			*–*		
*Men*	*914,957*	*100*	*–*			*1.8*			*–*			*1623*			*–*		
*Total*	*1,829,956*	*100*	*–*			*3.0*			*–*			*5469*			*–*		

^l^jobs at high risk of CTS; ^m^2009 INSEE Census data; a. % of the whole regional population; b. Standardized incidence ratios of CTS (SIR_job-cts_); c. 95% confidence interval (CI); d. Incidence of CTS per 1000 persons-years (I_job-cts_); e. Range computed using the lower and upper limits of the 95% CI of SIR_job-cts_; f. Attributable fractions of CTS (AFEjob-cts(%)); g. Range calculated using the lower and upper limits of the 95% CI of aRR_job-cts_ (data not shown); h. Total number of CTS (N_job-cts_) in the job considered; i. Range computed using the number of workers of each job and gender (N) and the lower and upper limits of the range of I_job-cts_; j. Number of WR-CTS cases (N_job-wr-cts_); k. Range computed using the lower and upper limits of the range of AFE_job-cts_(%)

Jobs labels: ‘Agriculture jobs’: skilled agricultural workers and laborers (PCS code 1) in the agriculture sector (NACE code 1); ‘Food industry jobs’: food and related products machine operators (PCS 67) in the meat and food industry sector (NACE 15), ‘Shoe industry jobs’: machine operators and assemblers (PCS 67) in the shoe industry sector (NACE 19); ‘Construction jobs’: building craft workers (PCS 63) in the construction sector (NACE 45); ‘Transport jobs’: drivers and mobile plant operators (PCS 64) in the transport sector (NACE 60); ‘Cleaning jobs’: personal service workers and cleaners (PCS 68) in the services to enterprises sector (NACE 74); ‘Trade jobs’: sales workers and cashiers (PCS 55) in the trade and commerce sectors (NACE 52); ‘Healthcare jobs’: government and public service personal care workers (PCS 52) in the health sector (NACE 85); ‘Personal services health jobs’: personal service workers (PCS 56) in the health sector (NACE 85)

Two jobs were at risk for both genders: food and related products machine operators in the meat and food industry sector (‘Food industry jobs’) and skilled agricultural workers and laborers in the agricultural sector (‘Agriculture jobs’).

Two jobs were at risk only in men: building craft workers (e.g., painters) in the construction sector (‘Construction jobs’) and drivers and mobile plant operators in the transport sector (‘Transport jobs’).

A total of 1603 [1137–2212] surgical CTS were estimated to have occurred in this subpopulation at risk, accounting for 29% of all cases registered in the region. Gender effect was observed with a greater number of CTS in women than in men (1265 [922–1702] vs. 338 [215–510]), accounting for 32% vs. 21% of all cases, respectively. A total of 906 [450–1522] cases were considered as attributable to work (WR-CTS) accounting for 57% of CTS in high-risk jobs (17% of all cases). Greater numbers of WR-CTS were also observed in women (664 [329–1106] vs 242 [121–416]), but the proportion of WR-CTS in comparison to CTS was lower than in men (53% vs 72%).

Large variations in CTS incidence and AFEs were observed in different jobs (Table 1). The three top jobs according to the number of CTS were ‘Healthcare jobs’, with 381 CTS, followed by ‘Trade jobs’ (288) and ‘Personal service health jobs’ (267). The three top jobs according to WR-CTS were the ‘Food industry jobs’ (195 cases) followed by ‘Healthcare jobs’ (183) and ‘Trade jobs’ (158).

As shown in Table 1, six jobs were characterized by high proportions of WR-CTS (> 60%) and fewer cases of CTS: ‘Agriculture jobs’, ‘Food industry jobs’, ‘Shoe industry jobs’, ‘Construction jobs’ ‘Transport jobs’ and ‘Cleaning jobs’. These six jobs (group 1) employing approx. 98,000 workers were associated with 740 [447–1274] CTS, including 563 [280–1101] WR-CTS (76%).

Three other jobs were characterized by moderate to low proportions of WR-CTS (< 60%) and large numbers of CTS (> 250): ‘Healthcare jobs’, ‘Trade jobs’ and ‘Personal service health jobs’. These jobs (group 2) employing approx.154,000 workers were associated with 936 [696–1290] CTS but only 406 [173–749] WR-CTS (43%).

### Estimated number of potentially preventable CTS cases in high-risk jobs

Greater numbers of CTS were preventable in women than men, in all the jobs and preventive scenarios considered. As shown in Table 2, the number of avoidable CTS varied between the different preventive scenarios and jobs.

**Table 2:** Estimated number of preventable cases of carpal tunnel syndrome (CTS) and preventive efficiency according the 10-WI, 5%-GI and 10%-GI preventive intervention scenarios

	*CTS N*_*job-cts*_^a^			Preventable CTS according to preventive scenarios^b^									Preventive efficiency^c^ (%)								
				10%-WI			5%-GI			10%GI			10%-WI			5%-GI			10%-GI		
	*N*	Range^d^		*N*	Range^e^		*N*	Range^f^		*N*	Range^f^		Mean	Range^g^		Mean	Range^h^		Mean	Range^h^	
‘Agriculture jobs’																					
Women^i^	82	61	108	6	4	9	4	3	5	8	6	11	7.3	3.7	14.8	4.9	2.8	8.2	9.8	5.6	18.0
Men^i^	34	20	54	2	1	4	2	1	3	3	2	5	5.9	1.9	20.0	5.9	1.9	15.0	8.8	3.7	25.0
Total	116	81	162	8	5	13	6	4	8	11	8	16	7.8	3.1	16.0	5.2	2.5	9.9	10.3	4.9	19.8
‘Food industry jobs’																					
Women^i^	152	98	225	13	7	20	8	5	11	15	10	23	8.6	3.1	20.4	5.3	2.2	11.2	9.9	4.4	23.5
Men^i^	78	45	127	7	4	12	4	2	6	8	5	13	9.0	3.1	26.7	5.1	1.6	13.3	10.3	3.9	28.9
Total	230	143	352	20	11	32	12	7	17	23	15	36	8.7	3.1	22.4	5.2	2.0	12.6	10.0	4.0	24.5
‘Shoe industry jobs’																					
Women^i^	16	11	22	1	1	2	1	1	1	2	1	2	6.3	4.5	18.2	6.3	4.5	9.1	12.5	4.5	18.2
Men	1	0	3	0	0	0	0	0	0	0	0	0		–			–			–	
Total	17	11	25	1	1	2	1	1	1	2	1	2	5.9	4.0	18.2	5.9	4.0	9.1	11.8	4.0	27.3
‘Construction jobs’																					
Women	22	1	124	2	0	12	1	0	6	2	0	12		–			–			–	
Men^i^	142	100	196	9	5	15	7	5	10	14	10	20	6.3	2.6	15.0	4.9	2.6	10.0	9.9	5.1	20.0
Total	164	101	320	11	5	27	8	5	16	16	10	32	6.7	1.6	26.7	4.9	1.6	15.8	9.8	3.1	31.7
‘Transport jobs’																					
Women	24	3	88	2	0	9	1	0	4	2	0	9		–			–			–	
Men^i^	84	50	133	6	3	11	4	3	7	8	5	13	7.1	2.3	22.0	4.8	2.3	14.0	9.5	3.8	26.0
Total	108	53	221	8	3	20	5	3	11	10	5	22	7.4	1.4	35.8	4.6	1.4	20.8	10.2	2.3	41.5
‘Cleaning jobs’																					
Women^i^	99	58	158	7	3	13	5	3	8	10	6	16	7.1	1.9	22.4	5.1	1.9	13.8	10.1	3.8	27.6
Men	6	0	36	0	0	4	0	0	2	1	0	4		–			–			–	
Total	105	58	194	7	3	17	5	3	10	11	6	20	7.6	1.5	29.3	4.8	1.5	17.2	10.5	3.1	32.8
*Group 1*																					
*Women*	*395*	*232*	*725*	*31*	*15*	*65*	*20*	*12*	*35*	*39*	*23*	*73*	*7.8*	*2.1*	*28.0*	*5.1*	*1.7*	*15.1*	*9.9*	*3.2*	*31.5*
*Men*	*345*	*215*	*549*	*24*	*13*	*46*	*17*	*11*	*28*	*34*	*22*	*55*	*7.0*	*2.4*	*21.4*	*4.9*	*2.0*	*13.0*	*9.9*	*4.0*	*25.6*
*Total*	*740*	*447*	*1274*	*55*	*28*	*111*	*37*	*23*	*63*	*73*	*45*	*128*	*7.7*	*2.2*	*24.6*	*5.0*	*1.8*	*14.3*	*10.1*	*3.5*	*28.4*
‘Trade jobs’																					
Women^i^	274	192	379	15	7	25	14	10	19	27	19	38	5.5	1.8	13.0	5.1	2.6	9.9	9.9	5.0	19.8
Men	14	2	49	1	0	5	1	0	2	1	0	5		–			–			–	
Total	288	194	428	16	7	30	15	10	21	28	19	43	5.6	1.6	15.5	4.9	2.3	10.8	10.1	4.4	22.2
‘Healthcare jobs’																					
Women^i^	381	302	475	18	11	28	19	15	24	38	30	48	4.7	2.3	9.3	5.0	3.2	7.9	10.0	6.3	15.9
Men	0				–			-,			-,			–			–			–	
Total	381	302	475	18	11	28	19	15	24	38	30	48	4.7	2.2	9.3	5.0	3.0	8.3	10.0	6.1	16.2
‘Personal services health jobs’																					
Women^i^	261	200	335	6	0	14	13	10	17	26	20	34	2.3	0.0	7.0	5.0	3.0	8.5	10.0	6.0	17.0
Men	6	0	35	1	0	4	0	0	2	1	0	4		–			–			–	
Total	267	200	370	7	0	18	13	10	19	27	20	38	2.6	0.0	8.5	4.9	2.7	9.5	10.1	5.4	18.5
*Group 2*																					
*Women*	*916*	*694*	*1189*	*39*	*18*	*67*	*46*	*35*	*60*	*91*	*69*	*120*	*4.3*	*1.5*	*9.7*	*5.0*	*2.9*	*8.6*	*9.9*	*5.8*	*17.3*
*Men*	*20*	*2*	*101*	*2*	*0*	*9*	*1*	*0*	*5*	*2*	*0*	*11*		–			–			–	
*Total*	*936*	*696*	*1290*	*41*	*18*	*76*	*47*	*35*	*65*	*93*	*69*	*131*	*4.4*	*1.4*	*10.8*	*4.9*	*2.7*	*9.3*	*10.0*	*5.3*	*18.5*
*All high-risk jobs*																					
*Women*	*1265*	*922*	*1702*	*66*	*33*	*111*	*64*	*47*	*85*	*126*	*92*	*172*	*5.2*	*1.9*	*12.0*	*5.1*	*2.8*	*9.2*	*10.0*	*5.4*	*18.7*
*Men*	*338*	*215*	*510*	*24*	*13*	*42*	*17*	*11*	*26*	*33*	*22*	*51*	*7.1*	*2.5*	*19.5*	*5.0*	*2.2*	*12.1*	*9.8*	*4.3*	*23.7*
*Total*	*1603*	*1137*	*2212*	*90*	*46*	*153*	*81*	*58*	*111*	*159*	*114*	*223*	*5.7*	*2.0*	*13.4*	*5.0*	*2.6*	*9.8*	*10.0*	*5.2*	*19.4*

^i^jobs at high risk of CTS; a. total number of CTS (N_job-cts_) in the job considered; b. *10% WI*: mono-component work-centered interventions targeting only work-related risk factors and expected to reduce WR-CTS cases by 10%; *5%-GI* and 1*0% GI:* multi-component global interventions targeting personal and work-related risk factors for CTS and expected to reduce both PR-CTS and WR-CTS by 5% or 10%, respectively;c. ratios of CTS hypothetically avoided / total number of CTS (%) in the job considered, computed only if one of the inferior range is superior than 0; d. Range computed using the number of workers of each job and gender (N) and the lower and upper limits of the range of incidence of CTS (I_job-cts_); e. Range calculated using the lower and upper limits of the range of the number of WR-CTS cases (N_job-wr-cts_); f. Range computed using the lower and upper limits of the range of N_job-cts_; g. Lower limit of range of PE = lower limit of range of WR-CTS hypothetically avoided / Upper limit of range of total number of WR-CTS and Upper limit of range of PE = Upper limit of range of WR-CTS hypothetically avoided / Lower limit of range of total number of WR-CTS; h. Lower limit of range of PE = lower limit of range of CTS hypothetically avoided / Upper limit of range of total number of CTS and Upper limit of range of PE = Upper limit of range of CTS hypothetically avoided / Lower limit of range of total number of CTS

Jobs labels: ‘Agriculture jobs’: skilled agricultural workers and laborers (PCS code 1) in the agriculture sector (NACE code 1); ‘Food industry jobs’: food and related products machine operators (PCS 67) in the meat and food industry sector (NACE 15), ‘Shoe industry jobs’: machine operators and assemblers (PCS 67) in the shoe industry sector (NACE 19); ‘Construction jobs’: building craft workers (PCS 63) in the construction sector (NACE 45); ‘Transport jobs’: drivers and mobile plant operators (PCS 64) in the transport sector (NACE 60); ‘Cleaning jobs’: personal service workers and cleaners (PCS 68) in the services to enterprises sector (NACE 74); ‘Trade jobs’: sales workers and cashiers (PCS 55) in the trade and commerce sectors (NACE 52); ‘Healthcare jobs’: government and public service personal care workers (PCS 52) in the health sector (NACE 85); ‘Personal services health jobs’: personal service workers (PCS 56) in the health sector (NACE 85)

Considering the jobs at risk overall (7 in women and 4 in men), the 10%-GI had the highest preventive efficiency, hypothetically preventing 159 [114–223] CTS out of 1603 [1137–2212] (PE =10.0% [5.2–19.4]). The 10%-WI prevented a lower number of cases than the 10%-GI (PE = 5.7% [2.0–13.4]), preventing at most 90 [46–153] cases, only slightly more than the 5%- GI, which avoided 81 [58–111] cases (PE = 5.0% [2.6–9.8]).

Considering the jobs targeted, the 10%-GI (PE = 10%) had maximum impact in the three jobs with the most cases of CTS: ‘Healthcare jobs’, ‘Trade jobs’ and ‘Personal service health jobs’, with 38, 28 and 27 cases prevented, respectively. The 10%-WI had maximum impact in the ‘Food industry jobs’ (20 cases avoided, PE = 8.7%), for which the preventive efficiency almost reached the level of the 10%-GI (23 cases avoided, PE = 10.0%). More generally, the 10%-WI was more efficient for jobs with high proportion of WR-CTS (PE = 7.7% for all the six jobs of group 1) than for jobs with moderate to low proportion of WR-CTS (PE = 4.4% for the three jobs of group 2) due to higher proportions of WR-CTS (76% vs 43%). Compared to the 5%-GI, the preventive efficiency of the 10%-WI was higher or at least equal for all jobs of group 1, but similar for ‘Healthcare jobs’ (PE = 4.8% vs. 5.0%) and ‘Trade jobs’ (PE = 5.5% vs. 5.1%), and even lower for ‘Personal services jobs’ (PE = 2.6% vs. 4.9%).

## Discussion

Most discussions on prevention strategies to reduce CTS have focused on reducing work-related risk factors in high-risk jobs. This study found that a limited proportion of CTS in a French general working-age population were work-related, and that work-related CTS were concentrated in several high-risk industries. This suggests that prevention efforts to reduce exposure to work-related risk factors should focus on high-risk jobs. Simulated workplace-based mono-component work-centered interventions and multi-component global interventions showed that preventive efficiency varied depending on the intervention design, the number of workers in different jobs and the proportion of work-related CTS. Given that personal risk factors such as diabetes and obesity are also risk factors for CTS, reducing rates of CTS in the general working-age population will also require strategies to reduce personal risk factors, particularly in jobs with low levels of work-related risk for CTS [20].

### Strengths and limitations

Surveillance data used for the computation of potentially preventable CTS included data from one of the largest and most complete surveillance programs for CTS, covering an entire region of France [1, 27].

The French PMSI database registering only surgical CTS underestimated cases potentially preventable since CTS requiring only medical treatment (e.g., corticosteroid injection) were not counted. The proportion of CTS requiring surgery is unknown in France. The surveillance of electrophysiologically confirmed CTS in the same region in 2002–2004 showed that about 66% of CTS did not undergo surgery (unpublished data). Analysis of compensation data in the Pays de la Loire region between 2008 and 2014 gave similar estimates: 63% (64% in men and 63% in women) of CTS compensated as occupational disease did not have undergone surgical treatment, without difference between the nine jobs at risk understudy (unpublished data). Surgical treatment is widely preferred to non-surgical or conservative therapies for overtly symptomatic patients, while mild cases are usually not treated, and therefore the surgical CTS included in the present study represented the most severe or most disabling cases of CTS in the regional population [30].

Given that the PMSI database lacked information relating to occupation and no more recent data were available, we used information on employment of patients undergoing surgery for CTS collected in 2002–2003 from the region’s three main hand surgery centers to estimate AFEs of CTS (unpublished data). No exhaustive job exposure data of the working population was available in the Pays de la Loire region, except the job titles collected by the 2009 Census. The use of job titles as surrogates variables for physical work exposure may result in significant exposure misclassification [31], but our estimates were in line with AFEs calculated in the only equivalent study comparing the incidence of surgical CTS in the Montreal region in 1995 [32]. Data from the pilot study described above revealed that the mean interval between the onset of the hand symptoms and the date of surgery was 3 to 4 years [1]. Therefore, AFE estimates probably reflected the working conditions in the late 90’s or early 00’s, and this may introduce bias in the calculation of the potential preventive efficiency of the preventive scenarios. Nevertheless, the SUMER French survey of working conditions conducted in 2003 and 2010 did not showed major changes of physical exposure in France, and it is unlikely that exposure to the main work-related risk factors for CTS drastically vary in the Pays de la Loire region [33].

Given that no more recent data were available that contained all needed variables, we analyzed surgical CTS registered in 2009 in the PMSI database and the 2009 census data to compute potentially avoidable surgical CTS according to different preventive scenarios. The incidence of surgical CTS was slightly higher in the Pays de la Loire region than in the whole France, but the region was characterized by a high employment rate. The incidence of surgical CTS had slightly decreased in the region since 2003 in men and above all in women, but we have no information indicating changes in surgeons’ practice and modification of the choice of surgical rather than conservative treatment of CTS in the region. French governmental action plans for improvement of occupational health were implemented in 2005–2009, but no clear conclusion can be drawn on the possible relationships between improved working conditions and decreasing trends in the incidence of CTS [1]. Finally, it is unlikely that higher decreasing incidence trends in women introduced major bias in the calculations of attributable fraction and potential preventive effectiveness, since our data were stratified according to gender.

The computation of AFEs took into consideration age and gender, which are the main unmodifiable personal risk factors for CTS, but not the possible joint effects of medical risk factors (e.g., obesity, rheumatoid arthritis and diabetes). Diabetes mellitus requiring pharmacological treatment (3.8% vs 4.2%) and obesity (13.3% vs 14.5%) were slightly less prevalent in the Pays de la Loire than in the whole France in 2009 [34, 35].

Certain very high-risk jobs involving few workers may not have been identified in the present study due to the lack of statistical power, and this might have led to underestimating the impact of work-centered prevention.

The computation of the preventable cases of CTS supposed several hypotheses [29], namely (i) causal relationships between the occurrence of CTS and work exposure and (ii) substantial impact of interventions reducing exposure to risk factors at the workplace [29]. Numerous biomechanical and epidemiological evidences argue in favor of causal relationships between biomechanical exposure at work and CTS [2, 8–10], even if the relative proportion of cases attributable to work is still under debate [2]. However, although decreasing exposure to work-related and/or personal risk factors was assumed to reduce the incidence of CTS by 10% in our study, evidence of such impact for primary prevention of CTS remains sparse, regardless of whether interventions will focus on personal factors [12, 13] or work-related factors [15–19, 22].

Information remains sparse on the impact of health promotion (such as weight loss) and/or specific exercises to prevent or reduce the incidence of CTS in the general working-age population [12, 13, 23]. We did not evaluate the hypothetical preventive efficiency of interventions that focus only on personal risk factors, expecting that changes in “personal risk factors” would be an essential component of multifaceted workplace interventions (10%-GI scenarios) [15]. Combining interventions on personal and work-related factors was assumed to have a higher impact than interventions targeting only on personal or work-related factors [15, 17]. To the best of our knowledge, we still lack data on the impact of multiple global interventions to estimate their joint effects. We have therefore adopted a simplistic additive model. We focused prevention only at the workplace level, although interventions to prevent CTS at the population level (e.g., media campaign of health promotion and prevention) might worth investigating.

### Interpretation

Most preventive interventions included in systematic reviews involve non-specific symptoms and focus on certain industry sectors (e.g., construction, healthcare) or occupational groups (e.g., office workers). Their effectiveness to decrease the incidence of CTS is still under debate [15–17, 22]. This hypothetical impact study conducted on surgical CTS at a regional population level showed that the potential preventive efficiency of workplace-based primary prevention of CTS will depend on (i) the theoretical efficiency of intervention, (ii) the targeting of jobs at the highest risk of CTS and (iii) the size of the population targeted. In practice, such interventions should be tailored by the professionals involved in the prevention and promotion of health at work.

Our study showed that the primary prevention of CTS had a greater impact on the number of preventable surgical CTS in women than men, regardless the jobs and scenarios of prevention considered. This was explained by the greater incidence of CTS in women leading to greater numbers of CTS [1], and concerned primarily PR-CTS rather than WR-CTS. Conversely, higher proportions of WR-CTS in men explained the slightly higher preventive efficiency of the 10%-WI in men. For ethical and legal reasons, workplace-based primary prevention should involve all workers exposed to occupational risks of CTS, regardless of gender.

Workplace-based interventions focusing on work-related risk factors had a greater impact on high-risk jobs and prevention efforts should focus on these jobs first. As expected, multiple global workplace-based interventions (10%-GI) were the most efficient strategy assuming additive effects on PR-CTS and WR-CTS. This is in line with systematic reviews reporting promising evidence for multifaceted interventions to prevent non-specific musculoskeletal disorders [15–17, 23]. However, we still lack of guidelines to implement such global multi-component interventions in real prevention practices [20, 23]. The highest preventive impact in our study concerned the two largest occupational groups at moderate risk of CTS (nursing aides and cashiers) and an occupational group at very high risk of CTS (operators in the meat and food industry). Focusing interventions on these three occupational groups would have the greatest impact on avoiding the majority of the preventable cases in the region.

## Conclusions

The prevention of CTS in the workforce remains a public health challenge [16]. This study using real surveillance data shows that the hypothetical preventive efficiency of workplace-based primary prevention of CTS will depend on (i) the theoretical efficiency of intervention, (ii) the level of risk and proportion of work-related cases in the jobs involved, and (iii) the size of the population or companies targeted. The impact of simulated workplace-based interventions suggests that prevention efforts to reduce exposure to work-related risk factors should focus mainly on high-risk jobs. Given that personal risk factors such as diabetes and obesity are also risk factors for CTS, reducing rates of CTS in the general working-age population will also require work-place based integrated strategies to reduce personal risk factors, particularly in jobs with low levels of work-related risk for CTS.

## Abbreviations

AFEs: Work-related attributable fraction of risk
aRR_*job-cts*_: Age-adjusted relative risks of CTS
CTS: Carpal tunnel syndrome
GI: Primary multi-component global interventions
I_*job-cts*_: Incidence rate of CTS in the job
I_*wp-cts*_: Incidence rates of CTS in the whole population
Nace: Classification of Economic Activities in the European Community
N_*e-job*_: Number of workers employed in the job
N_*job-cts*_: Total number of CTS in the job
N_*job-wr-cts*_: Number of WR-CTS cases in the job
PCS: French classification of occupations
PE: Preventive efficiency
PI: Personal intervention
PMSI: French National Medical Information Systems Program
PR-CTS: Personal-related cases of CTS
SIR_*job-cts*_: Standardized incidence ratios of CTS
WHO ICD-10: International Classification of Disease – World health Organization
WI: Work-centered ergonomic intervention
WR-CTS: Work-related cases of CTS
